# Trends in cancer incidence in Kyadondo County, Uganda, 1960–1997

**DOI:** 10.1054/bjoc.1999.1071

**Published:** 2000-04-03

**Authors:** H R Wabinga, D M Parkin, F Wabwire-Mangen, S Nambooze

**Affiliations:** Department of Pathology, Makerere University, PO Box 7072, Kampala, Uganda; The International Agency for Research on Cancer, 150 cours Albert Thomas, Lyon Cedex 08, 69372, France; Institute of Public Health, Makerere University, PO Box 7072, Kampala, Uganda

**Keywords:** cancer registry, time trends, Africa, AIDS

## Abstract

Incidence rates of different cancers have been calculated for the population of Kyadondo County (Kampala, Uganda) for four time periods (1960–1966; 1967–1971; 1991–1994; 1995–1997), spanning 38 years in total. The period coincides with marked social and lifestyle changes and with the emergence of the AIDS epidemic. Most cancers have increased in incidence over time, the only exceptions being cancers of the bladder and penis. Apart from these, the most common cancers in the early years were cervix, oesophagus and liver; all three have remained common, with the first two showing quite marked increases in incidence, as have cancers of the breast and prostate. These changes have been overshadowed by the dramatic effects of the AIDS epidemic, with Kaposi's sarcoma emerging as the most common cancer in both sexes in the 1990s, and a large increase in incidence of squamous cell cancers of the conjunctiva. In the most recent period, there also seems to have been an increase in the incidence of non-Hodgkin lymphomas. So far, lung cancer remains rare. Cancer control in Uganda, as elsewhere in sub-Saharan Africa, faces a threefold challenge. With little improvement in the incidence of cancers associated with infection and poverty (liver, cervix, oesophagus), it must face the burden of AIDS-associated cancers, while coping with the emergence of cancers associated with Westernization of lifestyles (large bowel, breast and prostate). © 2000 Cancer Research Campaign


*British Journal of Cancer* (2000) **82**(9), 1585–1592
© 2000 Cancer Research Campaign
DOI: 10.1054/ bjoc.1999.1071, available online at http://www.idealibrary.com on IDEAL®

### Trends in cancer incidence in Kyadondo County, Uganda, 1960–1997

**HR Wabinga[1], DM Parkin[2], F Wabwire-Mangen[3] and S Nambooze[1]**

[1]Department of Pathology, Makerere University, PO Box 7072, Kampala, Uganda; [2]The International Agency for Research on Cancer, 150 cours Albert Thomas, 69372 Lyon Cedex 08, France; [3]Institute of Public Health, Makerere University, PO Box 7072, Kampala, Uganda

**Summary** Incidence rates of different cancers have been calculated for the population of Kyadondo County (Kampala, Uganda) for four time periods (1960–1966; 1967–1971; 1991–1994; 1995–1997), spanning 38 years in total. The period coincides with marked social and lifestyle changes and with the emergence of the AIDS epidemic. Most cancers have increased in incidence over time, the only exceptions being cancers of the bladder and penis. Apart from these, the most common cancers in the early years were cervix, oesophagus and liver; all three have remained common, with the first two showing quite marked increases in incidence, as have cancers of the breast and prostate. These changes have been overshadowed by the dramatic effects of the AIDS epidemic, with Kaposi's sarcoma emerging as the most common cancer in both sexes in the 1990s, and a large increase in incidence of squamous cell cancers of the conjunctiva. In the most recent period, there also seems to have been an increase in the incidence of non-Hodgkin lymphomas. So far, lung cancer remains rare. Cancer control in Uganda, as elsewhere in sub-Saharan Africa, faces a threefold challenge. With little improvement in the incidence of cancers associated with infection and poverty (liver, cervix, oesophagus), it must face the burden of AIDS-associated cancers, while coping with the emergence of cancers associated with Westernization of lifestyles (large bowel, breast and prostate). © 2000 Cancer Research Campaign

**Keywords**: cancer registry; time trends; Africa; AIDS

Kampala Cancer Registry was established in 1954 with the aim of obtaining information on cancer occurrence in the population of Kyadondo County in which the capital city of Kampala is situated (Davies et al, 1965). The registry is located in the Department of Pathology in Makerere University Medical School. It functioned continuously both pre- and post-independence (1962), until the coup d'état of General Amin Dada in 1971. Thereafter, full population coverage was not possible, although a register was maintained within the Pathology Department until 1980, when all registration ceased. With the return of political stability, the registry was restarted (in 1989) and has functioned continuously since. This long period of operation provides a unique opportunity to study temporal changes in cancer patterns in an African setting. Within Kyadondo, there have been quite marked social changes over the last 40 years. Progressive urbanization of the population has meant that, although the total population has increased more than fourfold, Kampala city has grown almost tenfold, from a population of 92 000 in 1959 to 870 000 in 1995, and now represents three-quarters of the county population. There has also been an increase in educational standards: the population of 10 years and above who have ever been to school increased among males from 4.7% in 1959 to 71.8% in 1991 and among females from 1.8% to 61.9%. In addition, Uganda is one of the countries most affected by the HIV epidemic, and therefore temporal changes reveal its effect on cancer occurrence in Africans.

*Received 18 June 1999*
*Revised 20 October 1999*
*Accepted 4 November 1999*

*Correspondence to:* DM Parkin

Previous incidence rates for Kyadondo County have been published for the periods 1954–1960 (Davies et al, 1962, 1965) and 1968–1970 (Templeton et al, 1972). In addition, a comprehensive descriptive analysis for the period 1964–1968, based on all registrations (not restricted to Kyadondo residents, and without calculation of incidence rates), was published as a monograph (Templeton, 1973). The first results from the revitalized registry for a 28-month period in 1989–1991 were published in 1993 (Wabinga et al, 1993). Here we review incidence rates for Kyadondo County for two extended periods, 1960–1971 and 1991–1997, with a focus on the trends in risk of different cancers both during and between these periods.

### MATERIALS AND METHODS

### Cancer records

In 1953 the request/result forms of the Department of Pathology were redesigned specifically to permit registration of cancers. Thus they contain demographic information on the patient such as name, age, sex, tribe and place of residence, as well as the source of the specimen and results of the examination. In addition to data collected in this way, tumour registrars have been employed to search for cancer cases admitted to, or treated in, the four main hospitals in Kampala (and more recently in the Uganda Hospice) and, for individuals resident in Kyadondo County, to extract somewhat more extensive information onto special notification forms.

Between 1954 and 1980, registration was manual apart from the period 1964–1968 when the data were transferred to punchcards (Templeton, 1973), which are no longer available, with details of all cancer cases identified being entered in a large 'register'. Since 1989 the registration process has been computerized, using the

Table 1 Average annual population at risk, Kyadondo County, Uganda, and percentage by age group and sex

| | 1960–1966 | | 1967–1971 | | 1991–1994 | | 1995–1997 | |
|---|---|---|---|---|---|---|---|---|
| | M | F | M | F | M | F | M | F |
| 0–14 | 30.8 | 39.0 | 34.3 | 43.5 | 40.6 | 43.5 | 40.6 | 43.5 |
| 15–44 | 57.9 | 50.9 | 55.8 | 47.6 | 52.4 | 50.0 | 52.4 | 50.0 |
| 45–55 | 5.9 | 4.8 | 5.1 | 4.2 | 4.0 | 3.2 | 4.0 | 3.2 |
| 55–64 | 2.9 | 2.8 | 2.5 | 2.2 | 1.6 | 1.7 | 1.6 | 1.7 |
| 65+ | 2.3 | 2.4 | 2.2 | 2.4 | 1.3 | 1.7 | 1.3 | 1.7 |
| Total | 152 000 | 116 500 | 225 400 | 185 400 | 526 900 | 546 600 | 577 200 | 592 400 |

CANREG system (Cooke, 1998) which, at the stage of data entry, prevents the use of non-existent codes and performs checks for internal consistency between variables. It also permits a search for potential duplicate registrations.

The records from the registers of 1960–1980 have also been entered into the CANREG system (the registers for 1954–1959 were no longer available, and had probably been removed from the country). Several duplicate entries were identified, as well as a considerable number of errors and missing information. Doubtful records were completed, corrected or deleted, after tracing the original archives in the records of the hospitals and the pathology department.

Tumour site and morphology were coded according to the second edition of the International Classification of Diseases for Oncology (Percy et al, 1990). For tabulation of results, these were converted to the 10th revision of the ICD (WHO, 1992) and to the International Classification for Childhood Cancer (Kramárová et al, 1997).

Since registration ceased to be population-based between 1972 and 1980, the data for this period were not used in the current analyses. With the availability of 8 full years' registration since the restart of the registry in late 1989, it was clear that the case finding for 1990 was inadequate and so the data for this year, included in a previous publication (Wabinga et al, 1993) were excluded. Otherwise, inspection of the annual number of registrations (and proportions of registrations based on histological examinations) showed a regular progression with time. We therefore chose to consider four time periods: 1960–1966 (7 years); 1967–1971 (5 years), 1991–1994 (4 years); 1995–1997 (3 years).

### Population data

Population censuses were performed in 1959, 1969 and 1991. For these years, the population of Kyadondo County was available by sex and 5-year age group. An estimate for 1995 was provided by the Department of Statistics of the Ministry of Finance and Economic Planning; this was a simple extrapolation of the 1991 result, assuming an annual growth in each age group of 2.61% for males and 2.30% for females. An interpolation between the census of 1959 and 1969 was prepared, assuming a constant rate of increase within age/sex groups, and projections for 1996 and 1997 were made, assuming the same growth rate as that provided for 1991–1995.

For the calculation of incidence rates in the four periods considered, we used: the estimated population for 1963, the census population of 1969 and the estimated average populations for 1991–1994 and 1995–1997. These are shown in Table 1.

### Statistical methods

Incidence rates for each of the four periods were calculated for 5-year age groups. Age-standardized rates (world standard population) and 95% confidence limits were calculated as described in Boyle and Parkin (1991). Differences between rates were tested by means of the Mantel–Haenzel test, stratifying for age in 5-year groups (Estève et al, 1994).

### RESULTS

During the 19 years of registration considered (1960–1971; 1991–1997) a total of 7312 cases (3576 male and 3736 female) were registered. Tables 2 and 3 show, for males and females respectively, the number of cases in each of the four time periods, by major cancer sites, together with the age-standardized incidence rates, and their standard errors. The statistical significance of the change in the incidence rate from the preceding period is shown.

In both sexes, the total incidence of cancer (all sites combined) increased across the four periods. The most dramatic increase between the decades of the 1960s and the 1990s is in the incidence of Kaposi's sarcoma (KS), especially for females, in whom KS was previously a rather rare disease (sex ratio almost 20:1) but in whom the incidence rate is now around half that of men. Figure 1 shows age-specific incidence rates for KS, for 1960–1971 and 1991–1997 in men and, for the latter period only, in women. In males, as well as the increase in incidence, the shape of the curve has changed: in the 1960s there was a progressive increase in risk with age, while in the 1990s there is a small peak in incidence in childhood, and a more marked one in the late 30s. In females, incidence in childhood is lower, and the peak incidence in adults is at ages 25–29. The mean age at diagnosis in the 1991–1997 period is significantly later ($P < 0.001$) in men than in women both for all cases (32.0 vs 27.1), and for cases in adults aged 15 or more (34.9 vs 29.8).

Other than KS, the cancers showing the most marked increases in incidence in males are cancers of the prostate and oesophagus. There have also been significant increases in rates for cancers of the large bowel and eye and, in the last 3 years, of non-Hodgkin lymphoma. Although the changes in incidence between the individual time periods are not statistically significant, the trends in incidence rates between 1960–1966 and 1995–1997 are highly significant ($P < 0.01$) for both stomach and lung cancers. Conversely, there have been significant decreases in the incidence rates of cancers of the bladder and penis, and of Hodgkin's disease and leukaemia. Liver cancer, which was the most common cancer of men in the 1960s, appears to have declined in incidence during the 1990s.

 © 2000 Cancer Research Campaign

**Table 2** Incidence of the major cancers in Kyadondo County, Uganda, in four time periods: males

| | 1960–1966 | | | 1967–1971 | | | 1991–1994 | | | 1995–1997 | | |
|---|---|---|---|---|---|---|---|---|---|---|---|---|
| | No. | ASR | (standard error) | No. | ASR | (standard error) | No. | ASR | (standard error) | No. | ASR | (standard error) |
| Nasopharynx | 3 | 0.3 | (0.2) | 9 | 1.8 | (0.7) | 11 | 0.7 | (0.2) | 26 | 2.3 | (0.6)[b] |
| Oesophagus | 8 | 1.7 | (0.6) | 25 | 5.1 | (1.1)[c] | 83 | 15.8 | (1.9)[c] | 68 | 13.0 | (1.8) |
| Stomach | 14 | 2.7 | (0.8) | 20 | 4.7 | (1.1) | 31 | 4.7 | (1.0) | 37 | 7.6 | (1.4) |
| Colon & rectum | 14 | 3.0 | (0.8) | 23 | 4.8 | (1.1) | 52 | 8.3 | (1.3)[a] | 38 | 6.8 | (1.4) |
| Liver | 44 | 6.0 | (1.1) | 75 | 11.7 | (1.6)[c] | 73 | 9.8 | (1.4)[d] | 41 | 5.9 | (1.2)[d] |
| Lung | 5 | 0.8 | (0.4) | 10 | 2.1 | (0.8) | 25 | 4.1 | (1.0) | 19 | 3.2 | (1.0) |
| Melanoma | 1 | 0.1 | (0.1) | 8 | 1.3 | (0.6)[a] | 7 | 1.5 | (0.7) | 7 | 1.1 | (0.5) |
| Skin | 26 | 3.7 | (0.8) | 27 | 4.3 | (0.9) | 18 | 2.5 | (0.7)[e] | 20 | 4.1 | (1.0) |
| Kaposi's sarcoma | 28 | 3.2 | (0.7) | 29 | 3.7 | (0.8) | 670 | 39.3 | (2.1)[c] | 513 | 39.3 | (2.3) |
| Prostate | 13 | 3.1 | (0.9) | 27 | 6.8 | (1.4)[a] | 113 | 26.3 | (2.6)[c] | 139 | 39.2 | (3.7)[a] |
| Penis | 29 | 5.5 | (1.1) | 30 | 6.3 | (1.2) | 18 | 2.9 | (0.8)[e] | 23 | 4.4 | (1.1) |
| Bladder | 24 | 5.2 | (1.1) | 27 | 5.9 | (1.2) | 13 | 2.5 | (0.8)[f] | 10 | 2.9 | (0.9) |
| Eye | 5 | 0.4 | (0.2) | 12 | 1.1 | (0.4) | 43 | 2.3 | (0.4)[a] | 47 | 3.0 | (0.6) |
| Hodgkin's disease | 16 | 1.9 | (0.6) | 17 | 1.7 | (0.5) | 8 | 0.8 | (0.4)[f] | 11 | 1.3 | (0.6) |
| NHL | 32 | 3.9 | (0.8) | 32 | 3.6 | (0.7) | 76 | 3.6 | (0.5) | 95 | 7.4 | (1.1)[b] |
| Leukaemia | 22 | 2.2 | (0.6) | 24 | 3.2 | (0.9) | 13 | 0.7 | (0.2)[f] | 16 | 1.1 | (0.3) |
| ALL | 352 | 54.2 | (3.3) | 478 | 81.2 | (4.3) | 1456 | 149.1 | (5.2) | 1290 | 166.6 | (6.2) |
| ALL (except KS) | 324 | 51.0 | | 449 | 77.5 | | 780 | 109.8 | | 777 | 127.3 | |

Significant increase since preceding period: [a]$P < 0.05$; [b]$P < 0.01$; [c]$P < 0.001$.Significant decrease since preceding period: [d]$P < 0.05$; [e]$P < 0.05$; [f]$P < 0.001$.

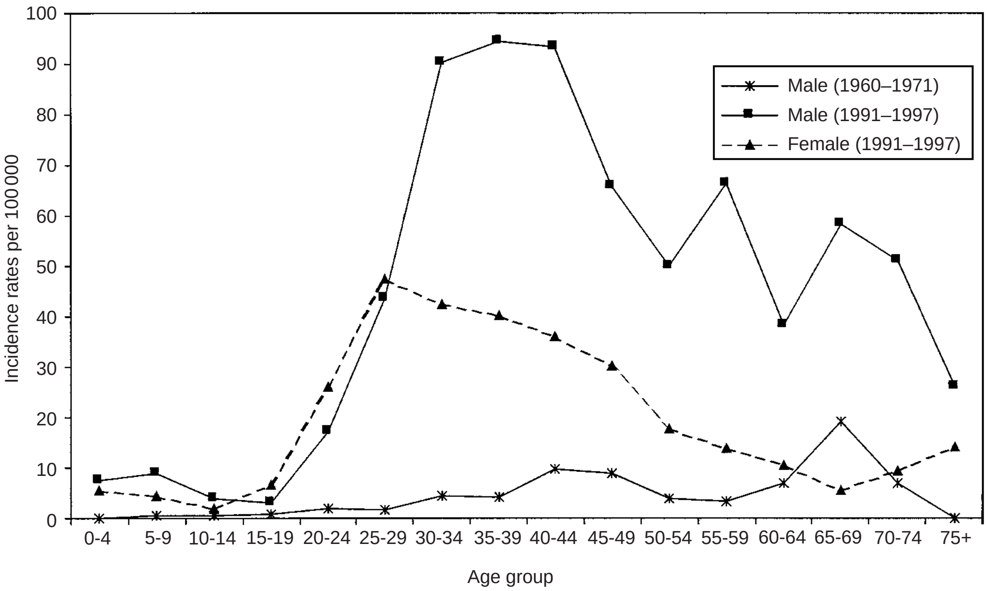

**Figure 1** Age-specific incidence rates of Kaposi's sarcoma

In females, the increasing trends for cancers of the oesophagus, stomach, large bowel, eye and non-Hodgkin lymphomas are similar to those in men, and there have also been increases in incidence of the two major cancers of women – breast cancer and cervix cancer. Figure 2 shows age-specific incidence rates of cervix cancer in the four different periods. The progressive increase of incidence with age in the earlier periods is replaced in the 1990s by a pattern of peak incidence around menopause, followed by a plateau. Nevertheless, there has been no significant alteration in the mean age at diagnosis between the four time periods (43.8, 42.8, 43.2 and 43.7).

The decline in bladder cancer incidence between the 1960s and 1990s has concerned almost exclusively squamous cell carcinomas (SCCs). In 1960–1971, 28/48 (58%) of histologically confirmed bladder cancer cases were SCCs, and just 10/48 (21%) transitional cell/adenocarcinomas. In 1991–1997 the percentages were 19% (3/16) SCC and 56% (9/16) transitional cell carcinomas.

Incidence rates of oesophageal cancer are similar in the two sexes, which is unusual for this tumour, even in the African continent. Forty-one per cent of the oesophageal cancers had information on histological subtypes. All were carcinomas, with 87.1% squamous, 7.1% adenocarcinomas and 5.7% unspecified subtype; there was no evidence of a change in the proportions over time.

The large increase in incidence of eye cancers is the consequence of increasing incidence of SCCs of conjunctiva. These increased from 4/17 eye cancers (23.5%) in men in 1960–1971

© 2000 Cancer Research Campaign

**Table 3** Incidence of the major cancers in Kyadondo County, Uganda, in four time periods: females

| | 1960–1966 | | | 1967–1971 | | | 1991–1994 | | | 1995–1997 | | |
|---|---|---|---|---|---|---|---|---|---|---|---|---|
| | No. | ASR | (standard error) | No. | ASR | (standard error) | No. | ASR | (standard error) | No. | ASR | (standard error) |
| Nasopharynx | 3 | 0.7 | (0.4) | 2 | 0.3 | (0.2) | 13 | 0.9 | (0.3) | 21 | 1.6 | (0.4)[a] |
| Oesophagus | 9 | 2.6 | (0.9) | 31 | 7.9 | (1.5)[c] | 55 | 9.4 | (1.3) | 63 | 14.2 | (1.9)[a] |
| Stomach | 4 | 0.8 | (0.5) | 14 | 3.4 | (1.0)[a] | 22 | 3.2 | (0.7) | 28 | 5.6 | (1.1) |
| Colon & rectum | 11 | 2.7 | (0.9) | 22 | 6.3 | (1.5)[a] | 36 | 5.7 | (1.1) | 34 | 6.6 | (1.2) |
| Liver | 9 | 1.8 | (0.6) | 21 | 5.0 | (1.3) | 42 | 5.1 | (1.0) | 35 | 6.3 | (1.3) |
| Lung | 3 | 0.6 | (0.4) | 6 | 1.4 | (0.6) | 7 | 0.7 | (0.3) | 18 | 3.2 | (0.9)[b] |
| Melanoma | 7 | 1.8 | (0.7) | 9 | 2.5 | (0.8) | 8 | 1.3 | (0.5) | 8 | 2.2 | (0.8) |
| Skin | 16 | 4.0 | (1.1) | 15 | 3.1 | (0.9) | 12 | 1.4 | (0.5)[d] | 8 | 1.0 | (0.4) |
| Kaposi's sarcoma | 1 | 0.1 | (0.1) | 2 | 0.2 | (0.1) | 360 | 17.9 | (1.2)[c] | 335 | 21.8 | (1.5) |
| Breast | 52 | 11.7 | (1.8) | 45 | 9.8 | (1.6) | 161 | 19.1 | (1.8)[c] | 146 | 22.0 | (2.1) |
| Cervix uteri | 84 | 17.7 | (2.2) | 109 | 22.5 | (2.5) | 341 | 39.7 | (2.5)[c] | 296 | 44.1 | (3.0) |
| Corpus uteri | 14 | 3.1 | (0.9) | 18 | 4.6 | (1.3) | 30 | 4.1 | (0.9) | 21 | 4.0 | (0.9) |
| Ovary | 26 | 5.7 | (1.3) | 19 | 3.2 | (0.8) | 62 | 6.9 | (1.1) | 41 | 5.3 | (1.0) |
| Vulva/vagina | 8 | 1.8 | (0.7) | 10 | 2.1 | (0.8) | 7 | 0.6 | (0.3) | 11 | 1.6 | (0.6) |
| Eye | 4 | 0.3 | (0.2) | 3 | 0.2 | (0.1) | 37 | 1.7 | (0.4)[c] | 45 | 3.4 | (0.7)[a] |
| Thyroid | 5 | 1.3 | (0.6) | 12 | 3.0 | (1.0) | 22 | 2.6 | (0.7) | 34 | 5.6 | (1.1) |
| Hodgkin's disease | 2 | 0.6 | (0.5) | 7 | 0.7 | (0.3) | 7 | 0.2 | (0.1) | 13 | 0.9 | (0.3) |
| NHL | 14 | 2.2 | (0.7) | 15 | 2.2 | (0.7) | 48 | 2.1 | (0.4) | 82 | 5.7 | (0.9)[c] |
| Leukaemia | 13 | 2.3 | (0.7) | 20 | 2.8 | (0.7) | 17 | 1.2 | (0.4)[f] | 17 | 1.9 | (0.6) |
| ALL | 338 | 73.0 | (4.4) | 469 | 98.9 | (5.3) | 1508 | 146.8 | (4.8) | 1421 | 179.7 | (6.0) |
| ALL (except KS) | 337 | 72.9 | | 467 | 98.7 | | 1148 | 128.9 | | 1086 | 157.9 | |

Significant increase since preceding period: [a]$P < 0.05$; [b]$P < 0.001$; [c]$P < 0.001$. Significant decrease since preceding period: [d]$P < 0.05$; [e]$P < 0.05$; [f]$P < 0.001$.

**Table 4** Cancer in children (age 0–14), Kyadondo County, 1960–1971 and 1991–1997

| | 1960–1971 | | | | 1991–1997 | | | |
|---|---|---|---|---|---|---|---|---|
| | *n* | (%) | M:F | Age standard rate (per $10^6$) | *n* | (%) | M:F | Age standard rate (per $10^6$) |
| Leukaemia | 25 | (18.4) | 1.1 | 18.7 | 27 | (4.9) | 0.7 | 8.6 |
| Hodgkin's disease | 11 | (8.1) | 2.7 | 8.7 | 9 | (1.6) | 1.3 | 2.8 |
| Burkitt's lymphoma | 13 | (9.6) | 1.6 | 9.5 | 109 | (19.7) | 1.5 | 34.3 |
| Other NHL[a] | 18 | (13.4) | 2.6 | 13.1 | 61 | (11.1) | 1.3 | 19.1 |
| Brain & CNS | 4 | (2.9) | 0.3 | 3.0 | 7 | (1.3) | 2.5 | 2.3 |
| Neuroblastoma | 5 | (3.7) | 1.5 | 2.9 | 1 | (0.2) | – | 0.3 |
| Retinoblastoma | 16 | (11.8) | 1.7 | 9.4 | 33 | (6.0) | 1.4 | 9.3 |
| Wilm's tumour | 10 | (7.4) | 0.7 | 6.1 | 29 | (5.3) | 1.6 | 8.6 |
| Osteosarcoma | 5 | (3.7) | 1.5 | 4.2 | 6 | (1.1) | 4.0 | 1.9 |
| Kaposi's sarcoma | 3 | (2.2) | 2.0 | 2.5 | 183 | (33.2) | 1.5 | 55.8 |
| Other STS | 10 | (7.4) | 4.0 | 7.6 | 20 | (3.6) | 0.8 | 6.0 |
| Carcinomas | 9 | (6.7) | 2.0 | 7.4 | 18 | (3.3) | 1.3 | 5.7 |
| Total | 136 | (100) | 1.6 | 97.8 | 552 | (100) | 1.4 | 169.7 |

[a]Includes lymphoma (unspecified).

(none of the seven eye cancers in women), to 32/45 (71% in men) and 34/40 (85%) in women in the most recent 3-year period (there were a few cases of unknown histological type).

Table 4 shows incidence rates for certain cancers of childhood (ages 0–14) in two periods, 1960–1971 and 1991–1997. The most striking change is the increased incidence of KS, from 2.5 per million in 1960–1971, to 55.1 per million in 1991–1997. The male predominance has persisted (sex ratio 1.5:1), and KS has become the most common tumour of childhood, ahead of the lymphomas. The mean age of these cases was 5.0, with no difference between the sexes, incidence was slightly higher in the age group 5–9 than at 0–4 (Figure 4). Lymphomas remain relatively frequent, with Burkitt's lymphoma (BL) by far the most frequently diagnosed in 1991–1997; the peak age is at 5–9 (Figure 3) with a mean age of 6.6, and boys predominate with a ratio of 1.4:1. The incidence of BL was significantly higher ($P < 0.001$) in the 1990s than in the 1960s. This is unlikely to be due to changing classification or coding, since the incidence of other and unspecified non-Hodgkin lymphomas was also higher in the second period (Table 4). In contrast, the incidence of childhood leukaemia is low, and there was a significant ($P < 0.05$) fall in incidence between the two periods.

### DISCUSSION

In order to study time trends, it is important that the degree of completeness of registration of incident cancer cases should be similar throughout the period under consideration. In recent years (since 1991) we believe that registration has been relatively good; an exercise in independent case ascertainment (Parkin et al, 1994),

 *© 2000 Cancer Research Campaign*

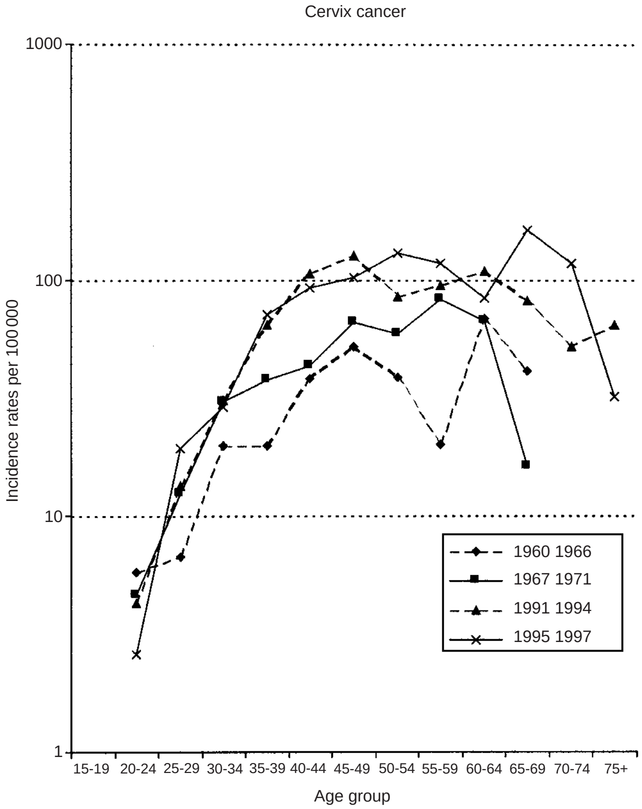

**Figure 2** Age-specific incidence rates of cancer of the cervix uteri

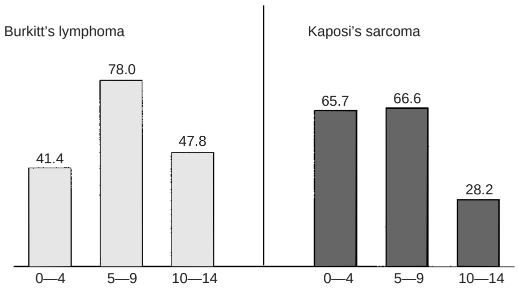

**Figure 3** Age-specific incidence rates for Burkitt's lymphoma and Kaposi's sarcoma of childhood in 1991–1997 (both sexes)

comparing data collected for epidemiological studies in Kampala with the registry database, suggested that the registry had identified 90% of incident cancers. For the earlier periods, we can be less certain.

Some indication of completeness of registration is also provided by the level of histological (or cytological) verification of registered cancer cases (Parkin et al, 1994). Table 5 shows that histological verification was considerably more frequent in the earlier time periods. This may be only a partial reflection of reality; certainly, before the country was plunged into political, social and economic chaos in the 1970s the standard of diagnostic (and therapeutic) services was higher than in the period of reconstruction in the early 1990s. But it is also possible that the case-finding mechanisms particularly in the first period studied here were less active than in the 1990s and that some cases without biopsies were missed. Finally, the wider availability of diagnostic modalities, such as endoscopy, in the 1990s may have improved case finding for certain cancers. The results should be interpreted, therefore, in the light of increases which could be due to better identification of cases diagnosed without histology. Templeton et al (1972) note that decreasing incidence rates are likely to be more meaningful than small increases in apparent incidence which might be related to improvements in diagnostic facilities.

**Table 5** Percentage of cases registered with diagnosis based on cytology or histology (MV%)

| | Period | | | |
|---|---|---|---|---|
| **Cancer site** | **1960–1966** | **1967–1971** | **1991–1994** | **1995–1997** |
| Oral cavity and pharynx | 91 | 97 | 59 | 84 |
| Oesophagus | 65 | 68 | 28 | 41 |
| Stomach | 83 | 74 | 30 | 57 |
| Colon and rectum | 86 | 87 | 58 | 69 |
| Liver | 85 | 79 | 33 | 36 |
| Lung | 75 | 81 | 41 | 76 |
| Kaposi's sarcoma | 76 | 94 | 78 | 86 |
| Breast | 82 | 88 | 56 | 68 |
| Cervix | 74 | 70 | 58 | 64 |
| Prostate | 62 | 67 | 66 | 80 |
| Bladder | 89 | 77 | 45 | 50 |
| Lymphoma | 96 | 93 | 72 | 80 |
| Leukaemia | 54 | 71 | 43 | 39 |
| All cancers | 84 | 84 | 64 | 73 |

In addition to these considerations, comparison of incidence rates requires that accurate population denominators are available. For Kyadondo, we had available estimates by age group and sex for three points during the long period studied, and made use of interpolations and projections for the other years. There must be some question as to the accuracy of the estimates of person-years at risk, therefore, particularly with respect to the age distributions, although no better data are, of course, available.

Davies et al (1964) reviewed hospital records from Mengo Hospital (in Kyadondo County) from 1897 to 1956. They noted that the general pattern of malignant diseases admitted to the hospital changed little over this period. The first estimation of incidence rates from the cancer registry of Kyadondo County was for 1954–1960 (Davies et al, 1962, 1965). The overall incidence reported for 1954–1960 was a little higher than our result for 1960–1966 (crude rates of 39.5 vs 33.1 for men and 48.4 vs 41.5 for women). It is possible that registration was rather more complete during the earlier period, when the proportion of cases registered without histology was 27.5%, compared with 16.4% in 1960–1966. On the other hand, experience with computer checks on the manual register for 1960–1971 showed that several duplicate registrations and non-malignant cases had been included, and these may also have inflated the numbers in the earlier time period. Nevertheless, for the individual cancer sites, the age-standardized rates are very similar in the two periods.

Incidence rates for the period 1967–1971 were very similar to those reported for 1968–1970 by Templeton et al (1972) with crude all-sites rates of 42.5 and 42.4 per 100 000 in men, and 50.2 and 50.6 per 100 000 in women.

Over the 38-year period, the cancers showing the most dramatic increases in incidence are those related to infection with HIV, particularly KS, and SCC of the conjunctiva (Beral and Newton, 1998). KS has always been observed in the Ugandan population,

*© 2000 Cancer Research Campaign*

although in the 1950s and 1960s it was of the typical ‘endemic’ pattern, involving the skin, particularly the legs, and affecting principally males, with the risk rising progressively with age (Taylor et al, 1971; Templeton, 1981). The enormous increase in incidence of KS since the earlier periods, together with the narrowing of the sex-ratio (from 18:1 in 1960–1971 to 1.7:1 in the 1990s) was noted in a previous report (Wabinga et al, 1993), and Ziegler and Katangole-Mbidde (1996) have drawn attention to the dramatic increase in the incidence of KS in children. Uganda, in common with other central African countries, has a very high prevalence of infection with HIV, and the age-specific incidence of KS corresponds closely to the age-specific reporting rates for AIDS, which are highest at ages 30–39 for men and 20–29 for women (UNAIDS, 1998). The prevalence of HIV infection among pregnant women had reached 30% in the urban population of Kampala in 1990–1992 (UNAIDS, 1998), but since that time it has been reported to be in decline (to around 15% in 1996–1997). This may explain the relative stability in the incidence of KS in 1995–1997 relative to 1991–1994 observed in this study. Currently, the evidence suggests that human herpes virus 8 (HHV8) is the aetiological agent responsible for KS (IARC, 1998). HHV8 has been identified in over 85% of KS tissue specimens in Uganda (Chang et al, 1996). Seroprevalence studies suggest a relatively high prevalence of infection by HHV8 in the general population of Uganda – considerably higher than in the USA and Europe, which would be consistent with the elevated frequency of ‘endemic’ KS which preceded the AIDS epidemic (Gao et al, 1996; Simpson et al, 1996).

Unlike KS, the incidence of non-Hodgkin lymphomas, which are found to be particularly common among immunosuppressed individuals in Europe and USA, had remained relatively stable in Kyadondo County in the early 1990s (Wabinga et al, 1993). However, in 1995–1997 there appears to have been a significant increase in incidence both in males and females. This may relate to improved survival of patients with HIV infection as other opportunistic infections are controlled, permitting a more prolonged duration of immunosuppression, and the development of more clinically-evident lymphomas.

Squamous cell carcinomas of the conjunctiva have been recognized for many years as more common in Africans than in Europeans, but there is also a considerable (tenfold or more) increase in risk in the presence of HIV infection (Newton, 1996). Ateenyi-Agaba (1995) has reported a large increase in the numbers of cases presenting clinically in Kampala since the onset of the AIDS epidemic.

The increase in the incidence of oesophageal cancer between the 1960s and 1990s, particularly among males, is difficult to explain. Studies in southern Africa, as elsewhere, have pointed to the importance of tobacco smoking and, less certainly, alcohol as being important in aetiology (van Rensburg et al, 1985; Segal et al, 1988; Vizcaino et al, 1995). The sex ratio of oesophageal cancer in Uganda has always been quite close to one to one, which suggests that tobacco and alcohol are not of major importance in the relatively high, and increasing, rates, since smoking (in particular) is largely confined to males. Furthermore, tobacco smoking is not common (average consumption per adult is 300 manufactured cigarettes per year), there has been little change between 1970 and 1990 (WHO, 1997), and the incidence of lung cancer has remained low in both sexes. Consumption of alcoholic beverages, on the other hand, is quite high (IARC, 1988) and after a decline through the 1970s to a low point of around 140 calories per caput per day in 1985, has since been increasing again (FAO, 1998). On the other hand, the importance of cereal-based diets, particularly those relying upon maize, has been stressed with respect to the risk in Africa, and in particular in explaining the marked differences in risk within quite small geographic areas (Cook, 1971; van Rensburg, 1981). The mechanism could be via nutritional deficiencies associated with such diets (van Rensburg et al, 1983; Jaskiewicz, 1989) or contamination of maize with mycotoxins (Sydenham et al, 1990). However, maize is not the staple food in Kyadondo area of Uganda, where consumption of a variety of plantains is the major source of carbohydrate.

The rather higher incidence rates of gastric cancer in the more recent periods possibly reflects better diagnosis because of the availability of gastroscopy; however, the incidence of this cancer remains low. This is certainly not due to a low prevalence of *Helicobacter pylori* – recognized by the IARC (1994) as an important cause – since the infection has been shown to be common, at least in the western part of Uganda (Wabinga, 1996). Tumours of the large bowel are also rare – an observation made by Burkitt 30 years ago (Burkitt, 1971), although there seems to have been something of an increase in incidence in both sexes since the 1950s and early 1960s, but little evidence of any recent change.

The reported rates of liver cancer in Uganda have remained low in comparison with other parts of sub-Saharan Africa although of course much higher than in Western populations. The reasons for this are not apparent. The prevalence of chronic carriage of hepatitis B in Uganda is similar to that in other countries, and aflatoxin contamination of foodstuffs appears to be common (Sebunya and Yourtee, 1990), though less perhaps in Kampala than in other parts of the country (Alpert et al, 1971).

The incidence rates of both breast and cervix cancer have approximately doubled between the 1950s and 1960s, and the 1990s. The reasons for these changes are not immediately clear. They are unlikely to be related to the epidemic of AIDS for, although cervix cancer is considered to be an AIDS-defining condition in the US, the evidence of a link between infection with HIV and the risk of invasive cervical cancer is not consistent. The role of oncogenic human papillomaviruses (HPV) in the aetiology of cervix cancer is clearly established (IARC, 1995). HPV was found to be present in the great majority of cervix cancers in Uganda, with HPV type 16 in 53% (Bosch et al, 1995). It seems quite plausible that the social disruption of the Amin dictatorship and following civil wars (1972–1986) had favoured the spread of HPV, like other sexually transmitted diseases. As far as breast cancer is concerned, it is possible that some of the increase is related to declines in fertility. Census data suggest that urban dwellers, and those with higher educational levels, have lower than average fertility, but it is unlikely that changes in these parameters could explain a doubling of incidence rates. Screening programmes for these cancers are virtually non-existent and can hardly have influenced trends. There has been virtually no change in the incidence of other female cancers – corpus uteri, ovary and vulva/vagina.

In men, the incidence of cancer of the prostate has increased remarkably, from an age-standardized rate of 3–6 per $10^5$ in the 1950s/60s, to 40 per $10^5$ in the late 1990s. This is one of the highest incidence rates recorded in Africa (Ferlay et al, 1998). Most of this increase is in elderly men – aged 65 or over – although the actual rates are not exceptionally high, compared with those reported in Europe and North America. The increase in Uganda is certainly not due to screening, although it is quite likely

 *© 2000 Cancer Research Campaign*

that increased awareness, a greater readiness to perform prostatectomy for urinary symptoms in elderly men, and histological examination of operative biopsies have played a role. The level of histological confirmation of diagnosis has actually increased over time (Table 3) in contrast to virtually all other sites.

In contrast to prostate cancer, the incidence of penile cancer in men is lower in the 1990s than in the 1960s (although the rates remain high – the age-standardized rate of 3–4 in Kampala, can be compared with 0.8 in the black population in the US SEER registries (Parkin et al, 1997)). Penile cancer was clearly very frequent in the early case series from Kampala (Davies et al, 1964), and Dodge et al (1973) found it 'the commonest tumour registered in males' in 1964–1968. This decline in incidence is probably real, since penile cancer is easily diagnosed and probably always brought for medical attention. Penile cancer has been related to genital hygiene (Kyalwazi, 1966) and the decline in incidence may be related to improved hygiene as a consequence of urbanization and greater availability of piped water supplies.

Bladder cancer is also significantly lower in the 1990s than in the 1960s. In Uganda, bladder cancer has been linked to the presence of urethral stricture. Dodge (1964) found 30% of bladder cancer causes had such strictures, and Owor (1975) found 4% of patients with strictures developed bladder cancer. Since strictures are a sequel to gonococcal infection, it is possible that better treatment for STDs may have reduced their prevalence. Bladder cancer is also a consequence of infection with *Schistosoma haematobium* (IARC, 1994). However, in Uganda *Schistosoma haematobium* has a rather restricted range and Kyadondo County is not among the endemic areas (Bradley et al, 1967).

### CONCLUSIONS

This long time series shows clearly the development of the problem of cancer in modern sub-Saharan Africa. New problems are emerging in the shape of AIDS-related cancers (KS, NHL, conjunctival neoplasms), and cancers associated with Westernization of lifestyles (breast and prostate cancers in particular, large bowel cancers less certainly). At the same time, there has been little, if any, decline in the major cancers of this region – cervix, liver and oesophagus. So far, Uganda, as most of the region, has been spared the epidemic of tobacco-related cancer that has been such an important feature of the cancer profile in economically developed countries. This reprieve is probably only temporary, however, and, while economic hardship may limit tobacco consumption and its health consequences, it will also restrict the capacity to mount effective programmes of cancer control.

### ACKNOWLEDGEMENTS

We acknowledge the cooperation and assistance of the medical nursing and records staff of Mulago, Rubaga, St Francis (Nsambya), and Mengo hospitals, and the Uganda Hospice, for their assistance in tracing and recording cancer patients, as well as the pathologists and haematologists for access to their laboratory records. We thank Ms Zam Kyalisiima for assistance in coding and data entry, and Mr Andy Cooke for help with the data analysis.

### REFERENCES

Alpert ME, Hutt MSR, Wogan GN and Davidson CS (1971) Association between aflatoxin content of food and hepatoma frequency in Uganda. *Cancer* **28**: 253–260

Ateenyi-Agaba C (1995) Conjunctival squamous-cell carcinoma associated with HIV infection in Kampala, Uganda. *Lancet* **345**: 695–696

Beral V and Newton R (1998) Overview of the epidemiology of immunodeficiency-associated cancers. *J Natl Cancer Inst Monogr*, pp. 1–6

Bosch FX, Manos MM, Munoz N, Sherman M, Jansen AM, Peto J, Schiffman MH, Moreno V, Kurman R and Shah KV (1995) Prevalence of human papillomavirus in cervical cancer: a worldwide perspective. International biological study on cervical cancer (IBSCC) Study Group. *J Natl Cancer Inst* **87**: 796–802

Boyle P, Parkin DM (1991) Statistical methods for registries In: *Cancer Registration: Principles and Methods*, Jensen OM, Parkin DM, MacLennan R, Muir CS and Skeet R (eds), pp. 126–158. IARC Scientific Publication No. 95. The International Agency for Research on Cancer: Lyon

Bradley DJ, Sturrock RF and Williams PN (1967) The circumstantial epidemiology of *Schistosoma haematobium* in Lango district, Uganda. *East Afr Med J* **44**: 193–204

Burkitt DP (1971) Epidemiology of cancer of the colon and rectum. *Cancer* **28**: 3–13

Chang Y, Ziegler J, Wabinga H, Katangole-Mbidde E, Bashoff C, Schultz T, Whitby D, Maddelena D, Jaffe HW, Weiss RA, the Uganda Kaposi's Sarcoma Study Group and Moore PS (1996) Kaposi's sarcoma-associated herpesvirus and Kaposi's sarcoma in Africa. *Arch Int Med* **156**: 202–204

Cook P (1971) Cancer of the oesophagus in Africa. *Br J Cancer* **25**: 853–880

Cooke A (1998) CANREG3 Manual. *Internal Report No 98/03*. International Agency for Research on Cancer: Lyon

Davies JNP, Wilson BA and Knowelden J (1962) Cancer incidence of the African population of Kyadondo (Uganda). *Lancet* **2**: 328–330

Davies JNP, Elmes S, Hutt MSR, Mtimavalye LAR, Owor R and Shaper L (1964) Cancer in an African community, 1897–1956. *Br Med J* **1**: 259–264

Davies JNP, Knowelden J and Wilson BA (1965) Incidence rates of cancer in Kyondondo county, Uganda 1954–1960. *J Natl Cancer Inst* **35**: 789–821

Dodge OG (1964) Tumours of the bladder and urethra associated with urinary retention in Ugandan Africans. *Cancer* **17**: 1433–1436

Dodge OG, Owor R and Templeton AC (1973) Tumours of the male genitalia. In: *Tumours in a Tropical Country. Recent Results in Cancer Control 41*, AC Templeton (ed). Springer: Berlin

Estève J, Benhamou E and Raymond L (eds) (1994) *Statistical Methods in Cancer Research. Vol IV, Descriptive Epidemiology*. IARC Scientific Publication No. 128. The International Agency for Research on Cancer: Lyon

FAO (1998) FAOSTAT Statistics Database: Food balance sheets (*http://apps.fao.org/*)

Ferlay J, Parkin DM and Pisani P (1998) *GLOBOCAN: Cancer Incidence and Mortality Worldwide*. IARC CancerBase No. 3. IARC: Lyon

Gao SJ, Kingsley L, Li M, Zheng W, Parravicini C, Ziegler J, Newton R, Rinaldo CR, Saah A, Phair J, Detels R, Chang Y and Moore PS (1996) KSHV antibodies among Americans, Italians and Ugandans with and without Kaposi's sarcoma. *Nat Med* **2**: 925–928

IARC (1988) *IARC Monograph on the Evaluation of Carcinogenic Risks to Humans, Vol 44, Alcohol Drinking*. International Agency for Research: Lyon

IARC (1994) *IARC Monograph on the Evaluation of Carcinogenic Risks to Humans, Volume 61, Schistosomes, Liver Flukes and* Helicobacter pylori. International Agency for Research: Lyon

IARC (1995) *IARC Monograph on the Evaluation of Carcinogenic Risks to Humans, Volume 64, Human Papillomaviruses*. International Agency for Research: Lyon

IARC (1998) *IARC Monograph on the Evaluation of Carcinogenic Risks to Humans, Vol 70, Epstein-Barr Virus and Kaposi's Sarcoma Herpesvirus/Human Herpesvirus 8*. International Agency for Research: Lyon

Jaskiewicz K (1989) Oesophageal carcinoma: cytopathology and nutritional aspects in aetiology. *Anticancer Res* **9**: 1847–1852

Kramárová E, Stiller CA, Ferlay J, Parkin DM, Draper GJ, Michaelis J, Neglia J and Quershi S (1997) *The International Classification of Childhood Cancer*. IARC Technical Report No. 29. International Agency for Research on Cancer: Lyon

Kyalwazi SK (1966) Carcinoma of the penis. A review of 153 patients admitted to Mulago Hospital, Kampala, Uganda. *East Afr Med J* **43**: 415–425

Newton R (1996) A review of the aetiology of squamous cell carcioma of the conjunctiva. *Br J Cancer* **74**: 1511–1513

Owor R (1975) Carcinoma of bladder and urethra in patients with urethral strictures. *East Afr Med J* **52**: 12–18

Parkin DM, Chen VW, Ferlay J, Galceran J, Storm HH and Whelan SL (1994) *Comparability and Quality Control in Cancer Registration*. IARC Technical Report No. 19. International Agency for Research on Cancer: Lyon

Parkin DM, Whelan SL, Ferlay J, Raymond L and Young J (Eds) (1997) *Cancer Incidence in Five Continents, Vol VII*. IARC Scientific Publication No 143. The International Agency for Research on Cancer: Lyon

*© 2000 Cancer Research Campaign*

Percy C, Van Holten V and Muir C (eds) (1990) *International Classification of Diseases for Oncology*, 2nd edn. World Health Organization; Geneva, Switzerland

Sebunya TK and Yourtee DM (1990) Aflatoxigenic Aspergilli in foods and feeds in Uganda. *J Food Quality* **13**: 97–197

Segal I, Reinach SG and de Beer M (1988) Factors associated with oesophageal cancer in Soweto, South Africa. *Br J Cancer* **58**: 681–686

Simpson GR, Schulz TF, Whitby D, Cook PM, Boshoff C, Rainbow L, Howard MR, Gao SJ, Bohenzky RA, Simmonds P, Lee C, de Ruiter A, Hatzakis A, Tedder RS, Weller IV, Weiss RA and Moore PS (1996) Prevalence of Kaposi's sarcoma associated herpesvirus infection measured by antibodies to recombinant capsid protein and latent immunofluorescence antigen. *Lancet* **348**: 1133–1138

Sydenham EW, Thiel PG, Marasas FO, Shephard GS, van Schalkwyk DJ and Koch KR (1990) Natural occurrence of some fusarium mycotoxins in corn from low and high esophageal cancer prevalence areas of the Transkei, Southern Africa. *J Agric Food Chem* **38**: 1900–1903

Taylor JF, Templeton AC, Vogel CL, Ziegler JL and Kyalwazi SK (1971) Kaposi's sarcoma in Uganda: a clinico-pathological study. *Int J Cancer* **8**: 122–135

Templeton AC (ed) (1973) *Tumours in a Tropical Country. Recent Results in Cancer Research*, No. 41. Springer Verlag: Berlin

Templeton AC (1981) Kaposi's sarcoma. *Pathol Ann* **16**: 315–336

Templeton AC, Buxton E and Bianchi A (1972) Cancer in Kyadondo County, Uganda, 1968–1970. *J Natl Cancer Inst* **48**: 865–874

UNAIDS (1998) Epidemiological fact sheet on HIV/AIDS and sexually transmitted diseases Uganda. http://www.who.int/emc-hiu/fact_sheets/

van Rensburg SJ (1981) Epidemiologic and dietary evidence for a specific nutritional predisposition to esophageal cancer. *J Natl Cancer Inst* **67**: 243–251

van Rensburg SJ, Ambrose SB, Rose EF and Plessis JP (1983) Nutritional status of African populations predisposed to esophageal cancer. *Nutr Cancer* **4**: 206–214

van Rensburg SJ, Bradshaw ES, Bradshaw D and Rose EF (1985) Oesophageal cancer in Zulu men. South Africa: a case–control study. *Br J Cancer* **51**: 399–405

Vizcaino AP, Parkin DM and Skinner MEG (1995) Risk factors associated with oesophageal cancer in Bulawayo, Zimbabwe. *Brit J Cancer* **72**: 769–773

Wabinga HR (1996) Frequency of *Helicobacter pylori* in gastroscopic biopsy of Ugandan Africans. *East Afr Med J* **73**: 691–693

Wabinga HR, Parkin DM, Wabwire-Mangen F and Mugerwa JW (1993) Cancer in Kampala, Uganda, in 1989–91: Changes in incidence in the era of AIDS. *Int J Cancer* **54**: 26–36

WHO (1992) *International Statistical Classification of Diseases and Related Health Problems, 10th Revision*. World Health Organization: Geneva, Switzerland

WHO (1997) *Tobacco or Health: A Global Status Report. Country Profiles by Region*. World Health Organization: Geneva, Switzerland

Ziegler JL and Katangole-Mbidde E (1996) Kaposi's sarcoma in childhood: an analysis of 100 cases from Uganda and relationship to HIV infection. *Int J Cancer* **65**: 200–203

*© 2000 Cancer Research Campaign*
